# Parasite Population Dynamics Shape *pfhrp2* and *pfhrp3* Deletion Patterns in the Peruvian Amazon

**DOI:** 10.21203/rs.3.rs-7255462/v1

**Published:** 2025-08-05

**Authors:** Erick Figueroa-Ildefonso, Luis Cabrera-Sosa, Johanna H. Kattenberg, Hugo O. Valdivia, Christopher Delgado-Ratto, Anna Rosanas-Urgell, Dionicia Gamboa

**Affiliations:** Laboratorio de Malaria: Parasitos y Vectores, Laboratorios de Investigación y Desarrollo, Facultad de Ciencias e Ingeniería, Universidad Peruana Cayetano Heredia; Grupo Malaria: Epidemiología Molecular, Unidad de Epidemiología Molecular, Instituto de Medicina Tropical “Alexander von Humboldt”, Universidad Peruana Cayetano Heredia; Department of Biomedical Sciences, Institute of Tropical Medicine; Department of Parasitology, U.S. Naval Medical Research Unit SOUTH (NAMRU SOUTH); Malaria Research group (MaRch), Global Health Institute, Family Medicine and Population Health department, Faculty of Medicine and Health Sciences, University of Antwerp; Department of Biomedical Sciences, Institute of Tropical Medicine; Laboratorio de Malaria: Parasitos y Vectores, Laboratorios de Investigación y Desarrollo, Facultad de Ciencias e Ingeniería, Universidad Peruana Cayetano Heredia

## Abstract

*Plasmodium falciparum* parasites with deletions of the *pfhrp2* and *pfhrp3* (*pfhrp2/3*) genes, involved in rapid diagnostic test (RDT) failure, have been increasingly predominant in the Peruvian Amazon since 2012. However, the evolutionary factors underlying this phenomenon remain unclear since HRP2-based RDTs have not been commonly used in this region. Here, we characterized the *P. falciparum* population in Peru (2006–2018) to identify genomic regions with evidence of recent positive selection. For this purpose, we PCR-genotyped 159 samples from the Loreto region, finding 60% with double *pfhrp2/3* deletions, 22% without deletions and 16% with single *pfhrp2* deletion. Then we performed whole genome sequencing (WGS) to a subset of the PCR-genotyped samples (n = 42) and integrated these results with existing genome data (n = 60). We revealed a significant reduction in the parasite population structure complexity from Period 1 (2006–2011) to Period 2 (2012–2018), suggesting a bottleneck event likely caused by the PAMAFRO control program. No selection signal was found on *pfhrp2/3* genes, supporting a population expansion of parasites carrying *pfhrp2/3* deletions due to genetic drift. Despite the clear change in phenotype (RDT evasion due to *pfhrp2/3* deletions), these results point to a different evolutionary direction than positive selection forces. This study also advocates the use of continuous surveillance using untargeted genomic approaches such as WGS to track emerging adaptations impacting malaria diagnostics and other control strategies.

## Introduction

Malaria is a tropical disease that remains a significant global health issue, particularly in endemic regions such as Peru. Despite intervention efforts by the Ministry of Health (MINSA), cases of malaria in Peru have been increasing in recent years. In 2024, a total of 33,314 cases were reported, representing the highest number of cases in the last 5 years, with approximately 95% of them concentrated in the Loreto region. *Plasmodium vivax* accounts for approximately 82% of malaria cases in the country, while *Plasmodium falciparum* represents around 18% of cases^1^.

Accurate and prompt diagnosis is a crucial factor in malaria control. The national elimination program in Peru, launched by MINSA in 2022, stipulates that the gold standard for malaria diagnosis is microscopy^2^. Main limitations of microscopy diagnosis are low sensitivity for low parasitemia infections and the need for specialized, well-trained personnel. An alternative diagnostic method is rapid diagnostic tests (RDTs), which detect parasite antigens and are valued for their ease of use in remote areas^3^. Nowadays, it is estimated that around 30% of malaria cases (representing around 10000 cases in 1000 communities in Loreto) are detected by RDTs^2^.

For *P. falciparum* diagnosis, RDTs primarily target histidine-rich protein 2 (HRP2), a protein abundantly secreted during the parasite’s blood stage and encoded by the *pfhrp2* gene. Additionally, histidine-rich protein 3 (HRP3), encoded by the *pfhrp3* gene, is structurally similar to HRP2 and can also be detected by HRP2-based RDTs^4^. During the Project for Malaria Control in Andean Border Areas (PAMAFRO), HRP2-based RDTs were first used in Loreto in 2007–2009^5^. However, since 2010, deletions of *pfhrp2/3* genes in *P. falciparum* parasites led to increasing concerns about false negatives RDTs results^6^, and HRP2-based RDTs were discontinued. Currently, RDTs based on lactate dehydrogenase (LDH) for *Plasmodium* are distributed in Peru.

The proportion of *phfrp2/3* deletions in Peru has changed through time. The prevalence of the *pfhrp2* deletion was moderate (21–41%) during the 2000–2010 period, and higher for *phrp3* (46–70%)^7–9^. However, since 2012, a significant increase in the prevalence of both *pfhrp2* and *pfhrp3* deletions (double deletion) was noted, reaching a prevalence of nearly 70% in 2014^10–12^. Even in indigenous, hard-to-reach communities, the deletions were present at a moderate proportion (32–50%) in 2020^13^. Beyond Peru, *pfhrp2/3* deletions have also been observed in Colombia (39%), Brazil (80%), Nigeria (13%), Sudan (11%), South Sudan (18%), Ethiopia (30%), among others^14–17^.

Factors driving the emergence and spread of parasites with *pfhrp2/3* deletions in the Peruvian Amazon remain unclear. In Africa, genomic analysis showed a recent selection pressure around *pfhrp2/3* genes, which favored parasites lacking *pfhrp2* due to the extensive use of RDTs, allowing them to evade detection and treatment^18,19^. However, this type of analysis has not yet been performed on *P. falciparum* populations in Peru, and particularly, the presence of any selective pressure in *pfhrp2/3* genes remains unclear.

Although microscopy is currently used as the main diagnostic tool in Peru, complemented with LDH-based RDTs, the PAMAFRO program might have created a similar scenario as observed in Africa, favoring parasites with *pfhrp2/3* deletions through intervention activities that initially used HRP2-based RDTs. In this context, this study aims to explore genomic regions that may have undergone recent positive selection in *P. falciparum* parasites from the Peruvian Amazon. By using genomic approaches, this study seeks to reveal potential genetic factors driving the increasing prevalence of *pfhrp2/3* deletions in Peru. This information could provide valuable insights into the genetic evolution of *P. falciparum* and its adaptation to their environment in the Peruvian Amazon.

## Methods

### Study design

This is an analytical observational study to genetically characterize the *P. falciparum* population in the Loreto region and identify selective sweeps, particularly around *pfhrp2/3* genes. First, we PCR-genotyped the *pfhrp2/3* deletions and their flanking genes in samples collected in 2013–2017 in Loreto (n = 159). Then, we generated whole genome sequencing (WGS) data for a sub-group of samples (n = 54) which was integrated with the only publicly available Peruvian genomic data at the time from the MalariaGEN consortium (n = 38, 2011–2018) and previously sequenced by our team (n = 54, 2006–2017), all from Loreto region too. This integrated genomic database was used for population genetic analysis and detection of signatures of selection.

### Sample collection and Ethics approval

Samples used for the PCR *pfhrp2/3* genotyping were collected in Loreto region from several previous studies (Supplementary Fig. S1). A longitudinal cohort study was conducted in Lupuna and Cahuide communities in 2013–2015 (n = 32); samples were collected by passive (PCD) and active (ACD) case detection. Also, as part of the WHO–FIND malaria RDT evaluation programs, ACD was conducted in Iquitos (2014) and San Juan Bautista (2017) districts (n = 3). Samples collected in Santa Emilia community by ACD and PCD came from two cohort studies (2013, n = 12 and 2015–2016, n = 47). Finally, as part of a passive surveillance study, samples were collected at Hospital de Apoyo de Iquitos (HAI) and Padre Cocha health center (2013–2016, n = 65). Participants came from several communities in Loreto, Maynas, Ramón Castilla y Datem del Marañón provinces.

Integrated genomes came from different available sources, including: the MalariaGEN consortium (samples from Iquitos, 2011 and Mazan, 2018 districts, n = 38)^20^, from the study published by Villena *et al*.^21^ (samples collected by PCD at HAI, 2006–2017, n = 18), genomic data previously generated by our team at UPCH in collaboration with the University of San Diego (UCSD) from samples collected by ACD in Nauta, Iquitos and San Juan districts (2015–2016, n = 13), and genomic data generated at the Institute of Tropical Medicine in Antwerp from samples collected as part of the WHO-FIND programs (2008–2010, n = 17).

All sample collections were approved by the Institutional Review Board (IRB) of Universidad Peruana Cayetano Heredia (UPCH) (SIDISI codes: 52707, 55587, 57395, 60429, 61703, 64024) and of the U.S Naval Medical Research Unit SOUTH (NAMRU-SOUTH) in compliance with all applicable federal regulations governing the protection of human subjects (protocol NMRCD.2007.0004). All participants and/or their legal guardians provided written informed consent during the study’s enrolment, which included a clause for future use of samples. This study received Ethical approval from the IRB of UPCH (SIDISI code: 205837).

### DNA extraction and parasitemia quantification

Genomic DNA was isolated using the EZNA^®^ blood DNA mini kit (Omega Bio-tek, Georgia, USA), following the manufacturer instructions, using 40 μL of whole blood or ~ 0.72 cm^2^ of dried blood filter paper samples and eluted in a final volume of 50 μL. *P. falciparum* parasitemia was quantified by a qPCR protocol targeting the 18S gene^22^.

### Detection of pfhrp2/3 deletions

Deletion of *pfhrp2/3* genes was detected by PCR amplification and results were visualized by agarose gel electrophoresis^13^. Quality control of DNA samples consisted in the amplification of the *glurp*, *msp1* and *msp2* genes^23^. Only samples that successfully amplified at least 2 out of 3 of these control genes were genotyped for *pfhrp2/3* deletions. Amplification of *pfhrp2/3* genes was conducted using primers targeting the exon 2 of both genes^13^. In a similar way, we performed the detection of their respective flanking genes PF3D7_0831900, PF3D7_0831700 (for *pfhrp2*), and PF3D7_1372400, PF3D7_1372100 (for *pfhrp3*)^11^.

We applied the Fisher’s exact test to compare the proportion of parasites with the deletions between Iquitos and Nauta districts (2013–2016), given that most samples were from those two places.

### Whole Genome Sequencing (WGS)

In order to compare parasite populations with and without *pfhrp2/3* deletions, genotyped samples with parasitemia > 200 parasites/uL were selected and separated in 2 groups according to their *pfhrp2/3* profile. In total, 54 *P. falciparum* samples (30 with *pfhrp2/3* deletion and 24 with both genes present) were chosen for WGS.

Then, we first selectively enriched the parasite’s genome using a set of primers as previously described^24^. The protocol consisted of two rounds of amplification using 1–20 ng of DNA quantified with the Qubit^™^ dsDNA HS Assay kit (Invitrogen, Massachusetts, USA) as starting input material. The amplification reaction mix was composed of 3.5 μM of the set of ten primers (set 6A and set 8A, for each round respectively), 30 units of phi29 DNA polymerase enzyme (New England Biolabs, Massachusetts, USA), 1mM of dNTPs and 0.1 mg/mL of bovine serum albumin, in a final reaction volume of 50 μL. The amplification protocol consisted of a temperature ramp from 35 to 30°C with a 10-minute step for each 1°C, followed by a 16-hour step at 30° and a final inactivation step at 65°C for 10 minutes, and was carried out in a T-100 thermocycler (Bio-Rad, California, USA). For the second round, we used 4 μL of the first-round product as input using the same reaction conditions as the first round. All amplified products were cleaned with the Ampure XP (Beckman Coulter, California, USA) beads system in a 1:1 ratio.

Library preparation was carried out using Nextera XT library preparation kit (Illumina, California, USA), following manufacturer instructions. The pooled libraries were sequenced in an Illumina MiSeq sequencer using the MiSeq reagent kit v2 (500 cycles) or v3 (600 cycles) to generate paired end reads between 250–300bp.

### Read mapping and variant calling

The reads of the 140 samples were processed to generate variant files (VCF). First, reads were trimmed with Trimmomatic, keeping reads with a minimum length of 50 bp, and quality parameters as recommended in the manual^25^. The trimmed reads were competitively mapped against the reference genome of the *P. falciparum* 3D7 (v3) and human (GRCh38.p13) reference genomes, using BWA-MEM version 0.7.17^26^. Variants were called using the HaplotypeCaller tool of GATK v4.2.6.1, followed by hard filtering of SNPs using recommended parameters proposed the GATK best practices^27^. Filters were set to keep SNPs with a minimum quality of depth of 2 (QD < 2), a minimum mapping quality of 40 (MQ < 40), and a minimum quality of 30 (QUAL < 30).

We excluded variants outside the “core” genome, with the exception of the *var* clusters regions, that were kept^28^. After a filtering step using VCF tools v0.1.16^29^, we kept only biallelic SNPs with < 25% of missing data and minimum average depth of 5X and finally removed samples with more than 25% of missing data.

### Determination of the pfhrp2/3 deletion using WGS data

As we did not perform PCR *pfhrp2/3* genotyping on the integrated available genomes, we used an *in-house* bioinformatic script to detect *pfhrp2/3* deletions in them. Briefly, we first calculated the mean coverage of each *pfhrp2/3* gene, of each exon 1 and 2 in each gen separately (using chromosomic positions available at PlasmoDB, https://plasmodb.org), and chromosome 8 and 13, considering all the positions in the bam file using bedtools. Then we calculated the ratio of the mean coverage for the whole *pfhrp2/3* gene, and each exon, to the mean coverage of the corresponding chromosome. Given that PCR for phrp2/3 genotyping targets exon 2 in each gene, for comparison purposes, we defined samples with deletion as the ones with a ratio of coverage for the exon 2 lower than 0.05.

### Complexity of infection determination

The Fws index was estimated with the moimix package in R^30^ and samples with Fws ≥ 0.95 were considered as monoclonal infections^31^.

### Population structure analysis

We pruned SNPs by Linkage disequilibrium (LD) using plink 1.9, keeping only SNPs with a r^2^ of 0.5 or lower^32^. For the Principal Component Analysis (PCA), we used the remaining SNPs after pruning, and we plotted the results with an *in-house* R script. The ancestry analysis of the samples was performed with Admixture v1.3.0^33^. We performed ten independent runs for values of K from 2 to 10 under a tenfold cross-validation procedure and 1000 bootstrap, using random values of initial seed for each K. We chose the most likely value of K considering the lower average cv-error for K. Using R-package pophelper^34^, we obtained the proportions and plot for the most probable number of clusters (K = 6).

### Nucleotide diversity

We determined the nucleotide diversity (*π*) by sliding across the target regions in the genome in 5000-bp windows using vcftools. To compare the median of π between Period 1 (2006–2011) and Period 2 (2012–2018), we performed the Kruskal-Wallis rank sum test.

### Site frequency spectrum (SFS)

To compare allele frequencies between Periods 1 and 2, we generated an unfolded 2D-site frequency spectrum (SFS) using the vcf2sfs and plot.sfs functions in R (https://github.com/shenglin-liu/vcf2sfs)^35^, and interpreted the plot as previously described^36^.

### Analyses to detect signatures of selection in Peruvian samples

Population structure showed that *P. falciparum* samples in Period 2 with and without *pfhrp2* gene belonged mainly to one subpopulation, different to each other. Because of that, all the tests to detect signatures of selection were applied in both these groups.

### Tajima’s D neutrality test

We used the python library scikit-allele 1.3.5^37^ (https://scikit-allel.readthedocs.io/en/stable/index.html) for Tajima’s D neutrality test^38^. The D statistic was estimated in 500 bp non-overlapping windows and was run separately for each chromosome. Since the statistics had a negative value across the genome, we only selected as outliers those regions with values of the D statistic equal or higher than 2.

### Linkage Disequilibrium-based tests

Each group of samples was phased using the default values of Shapeit 2^39^. We estimated the Integrated haplotype standardized (iHS) score to identify regions of the genome under positive selection. We used the R package rehh 3.2.2 to estimate the iHS score^40^. We highlighted those loci with |iHS| >3.29 as previously described^41^. A locus was assigned as candidate for being under selection, when −Log_10_(pvalue) ≥ 5 or if the |iHS| ≥ 4 and −Log_10_(p-value) ≥ 4.

We also compared the genomes grouped by carrying the deletion of the *pfhrp2* gene against those non-deleted. For that we performed the XP-EHH test included in the rehh R package^40,42^. A candidate locus was identified if the −Log10(p-value) ≥ 5.

### Gene Ontology (GO) Enrichment analysis

We enlisted the genes identified in the candidate loci under selection according for each group of parasites. Then, to test whether the gene sets were enriched for a molecular function in the parasite, we used the GO enrichment tool in PlasmoDB (https://plasmodb.org/plasmo/app/record/dataset/DS_fba620f00d)^43,44^.

## Results

### Profile of the pfhrp2/3 deletions with PCR genotyping

**Figure 1:**
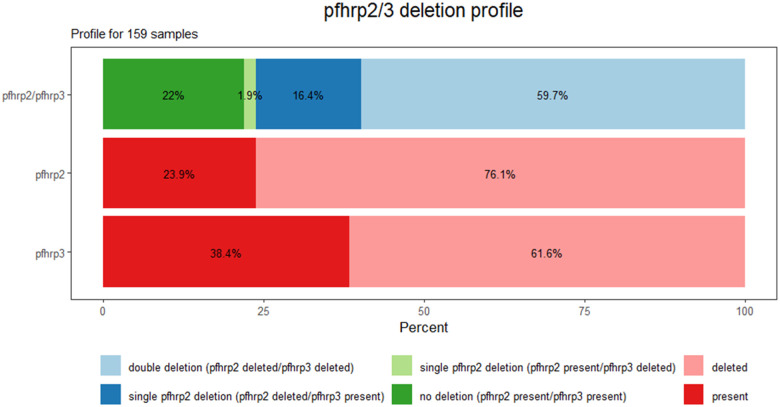
Profile of *pfhrp2* and *pfhrp3* gene deletions. The profiles of 159 samples genotyped by PCR were obtained by each gene separately and by taking both genes simultaneously.

We successfully PCR-genotyped 159 samples collected between 2013–2017 in Iquitos (n = 65), Nauta (n = 59), and nine other districts (Supplementary Table S1). Double *pfhrp2/3* deletion was the most common profile (95/159, 59,7%), followed by no deletions at all (35/159, 22%). The frequency of the *pfhrp2* and *pfhrp3* deletions (in single or double deletion) was 76.1% and 61.6%, respectively (Fig. 1). In samples with *pfhrp2*-deletetion, the flanking gene PF3D7_0831900 was also deleted in 95% (115/121) of samples. Similarly, the flanking gene PF3D7_1372100 was deleted in 93% (91/98) of the samples carrying the *pfhrp3*-deletion (Supplementary Table S2).

Because most samples were collected in Iquitos and Nauta districts during 2013–2016 (124/159, 78%), we determined the proportions of the deletion profiles in each district. Different profiles were predominant in each district (p < 0.0001). Double *pfhrp2/3* deletion (83%) was the most prevalent pattern in Iquitos. In contrast, double deletion was only at 19% in Nauta, where the single *pfhrp2* deletion (44%) was the most frequent profile (Supplementary Fig. S2).

### Profile of the pfhrp2/3 deletions and complexity of infection determined from genomic data

Genomic read data from 140 samples (54 samples from the PCR genotyping + 86 available genomes) were mapped to the 3D7 *P. falciparum* reference genome, enabling high quality genome-wide SNP calling and analysis for 102 samples and 38.180 biallelic SNPs after the quality controls described in the Methods section.

The complexity of infection was assessed using the Fws index. The median Fws was 0.95 (range: 0.75–1), indicating a predominance of monoclonal infections (Supplementary Fig. S3). Only 3 samples (2.9%) were classified as polyclonal infections.

We obtained the deletion profile for 102 samples using the coverage ratio of exon 2 for the *pfhrp2* and pfrhp3 genes (Supplementary Table S3). The double *pfhrp2/3* deletion was the most common profile (53/102, 52%), followed by no deletions (25/102, 24.5%). Among samples with PCR results (n = 42), all samples except 2 had a matching deletion profile between both methods (WGS and PCR); the two exceptions had a *pfhrp3* deletion by WGS, but not by PCR.

### Temporal and pfhrp2-based differentiation of P. falciparum populations

**Figure 2:**
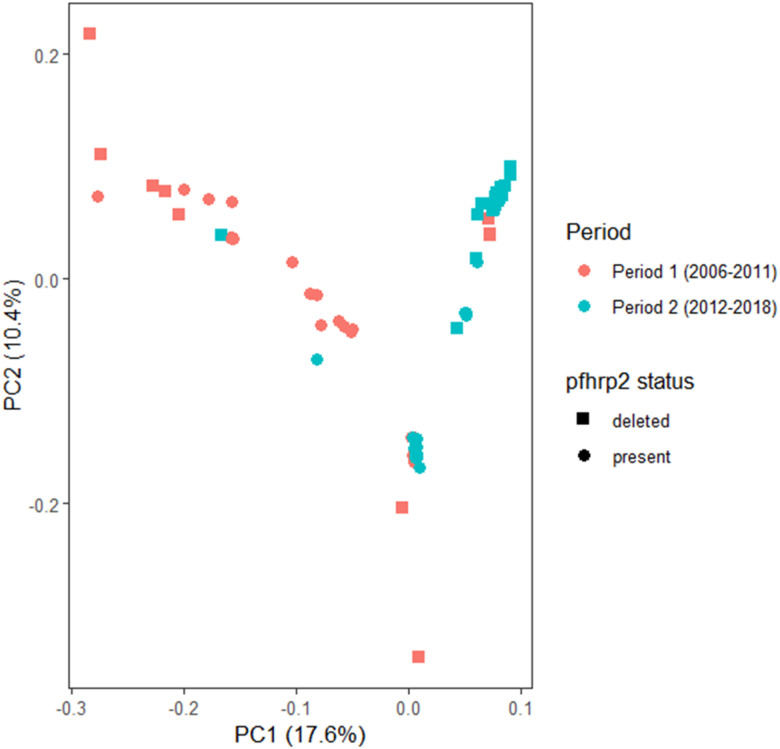
Principal component analysis (PCA) of 102 *P. falciparum* samples from Peru (2006–2018). Samples were identified according to the period of collection (color: Period 1: 2006–2011; Period 2: 2012–2018) and the presence or deletion of the *pfhrp2* gene (shape).

We observed a temporal differentiation in two periods of *P. falciparum* populations from Peruvian Amazon (Fig. 2). Most samples collected between 2006–2011 (Period 1) were separated from those collected in later years (Period 2, 2012–2018). In addition, samples collected in Period 2 were differentiated between samples carrying *pfhrp2* deletion from those without the deletion (Fig. 2). On the other hand, there was no clustering pattern due to the geographical origin of samples (Supplementary Fig. S4).

**Figure 3:**
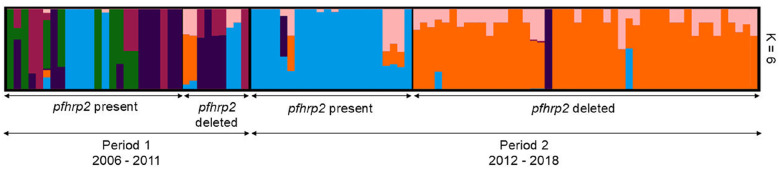
Admixture analysis of 102 *P. falciparum* samples from Peru (2006–2018). The graph depicts the clustering when the isolates were assigned into K=6 different clusters. Each isolate is represented by a single vertical line broken into 6 colored segments, with lengths proportional to each of the inferred clusters. Samples were grouped according to the period of collection (Period 1: 2006–2011, Period 2: 2012–2018) and the presence or absence of the *pfhrp2* gene.

Admixture analysis showed a reduction in the number of *P. falciparum* populations between the two periods (Fig. 3). Based on the cross-validation error, the most probable number of clusters in the parasite population was K = 6 (Supplementary Fig. S5). When plotted and grouped according to time and *pfhrp2* deletion status, we observed a clear change in the parasite population complexity. Samples from Period 1 (2006–2011) were admixed, belonging to 6 populations, regardless of their *pfhrp2* deletion status. In contrast, in Period 2 (2012–2018), admixture was also present, but with a smaller number of populations than Period 1 (6 to 3). Also, parasites with and without the *pfhrp2* deletion belonged mainly to 2 different populations (light blue and orange, respectively) (Fig. 3).

To discard any geographical influence in these findings, a subanalysis with only samples from Iquitos (n = 44) and Nauta (n = 17) districts was carried out. The PCA showed differentiation between both Periods, regardless of the district of collection. Also, the admixture analysis showed a reduction in the number of populations from Period 1 to Period 2, with parasites with and without *pfhrp2* deletion in Period 2 belonging to 2 main different populations (Supplementary Fig. S6).

We compared the nucleotide diversity (π) between both periods and noted a significant reduction in the genetic diversity (π-Period 1 = 1.8×10^− 4^, π-Period 2 = 7.8×10^− 5^, p < 2.2×10^− 16^) from Period 1 to Period 2 (Supplementary Fig. S7). We also compared the allelic frequencies using a 2D-site frequency spectrum (SFS). High counts of low-frequency variants were observed around the x-axis, suggesting a genetic drift in Period 2. Also, horizontal count bands crossing in the middle of y-axis represent allele sharing resembling the history of both periods (Supplementary Fig. S8).

### Absence of signatures of selection of the pfhrp2 and pfhrp3 gene deletions

**Figure 4:**
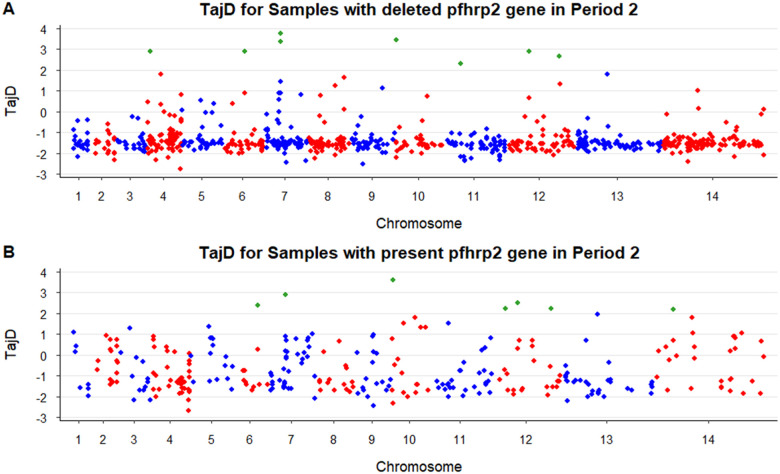
Manhattan plot of Tajima’s D neutrality test in *P. falciparum* from Peru in Period 2 (2012–2018). Significant values (TajD > 2) are indicated in green. Tajima’s D neutrality test was performed within (A) parasites with the *pfhrp2* deletion (n=47) or (B) parasites carrying the *pfhrp2* gene (n=22).

We investigated the presence of selective sweeps in the parasites from Period 2 (2012–2018) (47 and 22 samples with and without *pfhrp2* deletion, respectively) because each group belonged to one main different population according to the admixture analysis (Fig. 3). No signal of selection was found in the genomic regions surrounding the *pfhrp2* and *pfhrp3* loci. The Tajima’s D neutrality test resulted in most negative values across the genome (Fig. 4, Supplementary Table S4). Outlier positive values were detected in both groups of parasites (with and without the *pfhrp2* gene) in chromosomes 6, 10 and 12, highlighting genes from the *var* family and *ACBP1* gene. Particularly, parasites with *pfhrp2* deletion presented significant values in a genomic region in chromosome 11, where the *FP2A* gene was identified.

**Figure 5:**
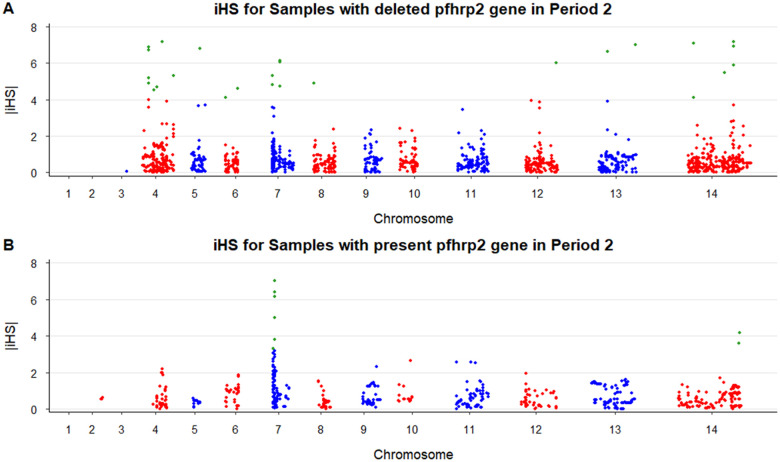
Manhattan plot of |iHS| in *P. falciparum* in Period 2 (2012–2018). Significant values (exceeding the threshold of 5 for −log10P-value, or that were considered of interest, having |iHS| ≥ 4 and −log10P-value ≥ 4) are indicated in green. iHS test was perfomed within (A) parasites with the *pfhrp2* deletion (n=47) or (B) parasites carrying the *pfhrp2*gene (n=22). Chromosomes and loci with no results for iHS were removed.

Similarly, none of the LD-based tests detected any significant signal around the *pfhrp2/3* genes. However, other genomic regions were identified under selection based in the |iHS| score (Fig. 5, Supplementary Table S5). Among parasites with *pfhrp2* deletion, signals were detected in chromosomes 4–8, and 12–14. From these signals, we identified some genes with known functions, including *rifin* in chromosome 4, var and *CDPK4* genes in chromosome 7, and AP2-G in chromosome 12. On the other hand, the *rifin* gene was the only identified with known function within the signals from parasites with *pfhrp2* gene present. The rest of the signals were in genes without defined functions or pseudogenes (Supplementary Table S5). Additionally, the XP-EHH analysis identified signals of selection in the *FP2A* gene, genes of the *var* family and other regions without defined function (Supplementary Fig. S9, Supplementary Table S6).

The false discovery rate (FDR) was evaluated for the GO terms of the candidate genes identified with signals for selection in both groups of parasites (with and without the *pfhrp2* deletion). Within parasites with deletion, GO terms of adhesion-related processes (cell adhesion, cell-cell adhesion) and chromatin organization (protein-DNA complex assembly) were significant (FDR = 0.003–0.04). Other terms related to host-parasite interaction and immune evasion showed elevated enrichment but were not significant (Supplementary Fig. S10, Supplementary Table S7). On the other hand, in parasites with *pfhrp2* gene, host interaction processes such as cell adhesion, evasion of the host immune response, response to other organisms and antigen evasion were significant (FDR < 0.01) (Supplementary Fig. S11, Supplementary Table S8).

## Discussion

Despite the high frequency of *pfhrp2/3* deletions in *P. falciparum* parasites in the Peruvian Amazon^10,12^, factors leading to the spread of these deletions are unclear. Here, we genomically characterized *P. falciparum* population and found a reduction of the complexity of the population structure during 2011–2012, but no recent signals of selection in the *pfhrp2/3* genes in parasites from Peru.

The population structure of *P. falciparum* in Peru has experienced a marked reduction in complexity over time, going from 6 parasite clusters in 2006–2011 (Period 1), in agreement with previous publications analyzing samples from 1998 to 2011^7,8^; to mainly 2 populations in 2012–2018 (Period 2), also in agreement with other reports^10,12,21^. These results strongly suggest that a bottleneck event significantly impacted the parasite population in the Peruvian Amazon around 2011, leading to the 2 populations, one with parasites with *pfhrp2/3* deletions and another without the deletions. The most plausible driver of this bottleneck was the sum of multiple intervention strategies (early diagnosis, treatment, and vector control measures) of the PAMAFRO control program, which operated in the Loreto region from October 2005 to December 2010^5,45^. Consistent with this hypothesis, we reported a reduction in genetic diversity from Period 1 to 2, and the SFS suggested the effect of genetic drift in the parasite population from Period 2. Also, the decline in malaria cases reported until 2011 coincides with reduced effective population size, genetic diversity and allelic richness in *P. falciparum*, as well as the reduced population structure complexity^10,12^.

As genetic drift can exacerbate the effects of a bottleneck, changes in allele frequencies occur randomly, leading to the fixation or loss of a genetic variant^46^. In our study, parasites with the *pfhrp2/3* deletions persisted after the bottleneck event due to PAMAFRO program, with the subsequent expansion of this population in the following years. This would explain the high frequency of *pfhrp2/3*-deleted parasites after 2012, coinciding with an increase in malaria cases until 2017, when MINSA launched a new national control program^47^. In agreement with this, we found that both *pfhrp2/3* genes were not under selection and even, no strong, uniform selection signals were found across the genome. Also, this “bottleneck followed by population expansion” phenomenon has been reported during early 2000s in the Peruvian *P. falciparum* population^48^. In that sense, genetic drift can explain better the evolution of the *pfhrp2/3* deletion rather than a selection event in this region. This contrasts with the recent report in Ethiopia, which showed evidence of the positive selection of phrp2 gene explained by the extensive use of RDTs^18^, highlighting that different evolutionary pathways can explain *pfhrp2/3* deletions expansion in different contexts.

Previous works have reported that Bv1 strain has been pivotal in the *P. falciparum* epidemiology in Peru since 2011^21,49^ and it is likely that Bv1 or similar strains have been expanding after the bottleneck^10,12^. This strain harbors many mutations associated with drug resistance and double *pfhrp2/3* deletion and has been involved in outbreaks in Loreto and other regions such as Tumbes and Cuzco^9,50^. Furthermore, hypotheses have been proposed to explain why parasites with *pfhrp2/3* deletions have been expanded over those with both genes present^12^. Hypotheses include lower immune response in the community against new strains and less competition and recombination due to low incidence. Also, parasites with deletions may show distinct phenotypes that can contribute to better fitness. For example, *in vitro* studies with strains like Dd2 (with single *pfhrp2* deletion) and 3D7 (without deletions) have revealed differences in the expression of genes related to invasion and heme metabolism^51,52^. Finally, *pfhrp2/3* deletions could arise to diagnostic evasion, particularly when HRP2-based RDTs were used in the PAMAFRO program^5^ and might have contributed as an advantage feature.

Although the signal was not strong, we identified some selective sweeps in genes associated with key parasite functions. For immune evasion, we observed signals in var genes, known to mediate immune evasion through antigenic variation^53^. In relation to sexual differentiation, the *AP2-G* gene – involved in sexual development at early stages^54,55^ – may contribute to parasite transmission, similar to previously reported for *gdv1* gene in Africa^56,57^. Regarding antimalarial drug response, signal were detected in *ACBP1* and *FP2A*, which encode an acyl-CoA binding protein and falcipain 2a, respectively; both may be involved with the parasite response to treatment, as mefloquine targets acyl-CoA interactions^58,59^ and falcipain 2a is required for potentiating artemisinin activity^60^. Whole-genome sequencing (WGS) approaches are essential to investigate hitchhiking – where the deletions are linked to other advantageous features within the same parasite, as they provide a comprehensive view of the parasite genome that methods like microsatellite (MS) genotyping or targeted analyses cannot offer^61,62^. However, because smaller populations (such as the low-transmission *P. falciparum* population in Peru) are more susceptible to the effects of genetic drift, results from selective sweep tests should be interpreted with caution^63^.

The proportion of parasites carrying the *pfhrp2* deletion has continued to increase since the first report in 2010^6^. We also report a high frequency of double *pfhrp2/3* deletion between 2013 and 2017, a pattern previously described in Peru^7,10,12^. In addition, different profiles were predominant in Iquitos and Nauta district, with the double deletion being most prevalent only in Iquitos. Nauta is a more remote and less accessible district than periurban Iquitos, and thus having limited gene flow (influx of new parasite populations, including the ones carrying the deletions). Also, the bottleneck event could have led to slightly different populations in these districts by chance, which may have persisted over time due to limited gene flow. Similarly, a high proportion of parasites with no deletion or single *pfhrp2* deletion has also been reported in an indigenous community in Trompeteros district, even more remote than Nauta^13^, highlighting the importance of surveillance of *pfhrp2/3* genes also in hard-to-reach areas.

The main limitation of this study is the heterogenous sample selection, including different origins, times of collection, study designs, sample sizes, etc, which may affect the interpretation and generalizability of the genomic analysis findings. As many results were supported by previous reports, a more systematic sampling approach is recommended. Although we conducted analyses supporting the bottleneck hypothesis, such as observing reductions in genetic diversity and shifts in the SFS, additional approaches, including simulations, could further complement and strengthen our findings^64,65^. Further works investigating the *pfhrp2/3* evolution in Peru should consider both suggestions.

In conclusion, our study demonstrates a significant reduction of *P. falciparum* population structure complexity around 2011 in Peru, most likely due to a bottleneck by the PAMAFRO program. Also, we found no selection signals in the *pfhrp2/3* genes, indicating that the population expansion of the parasites carrying the *pfhrp2/3* deletions in Peru may be explained due to genetic drift rather than positive selection. Together, these insights contribute to a deeper understanding of the genetic evolution of *P. falciparum* in the Peruvian Amazon and highlight the importance of genomic approaches to unravel the *pfhrp2/3* deletion dynamics.

## Supplementary Files

This is a list of supplementary files associated with this preprint. Click to download.

- SupplementaryFigures.pdf
- SupplementaryTables.xlsx

## Data Availability

Sample metadata, locations, dates and *pfhrp2* and *pfhrp3* profiles are accessible in the Supplementary Table 1.New raw data (FASTQ files) are available at the SRA under BioProject accession number PRJNA1289377. Some genomic data was taken from BioProjects PRJEB2136 and PRJEB46168. All individual library accession numbers are listed in the Supplementary Table S3.
